# Self-medication practice and associated factors among adult household members in Meket district, Northeast Ethiopia, 2017

**DOI:** 10.1186/s40360-018-0205-6

**Published:** 2018-04-10

**Authors:** Aster Desalew Kassie, Berhanu Boru Bifftu, Habtamu Sewunet Mekonnen

**Affiliations:** 1 Boru Meda District Hospital, South Wollo Zone, Amhara Region, Southeast Ethiopia; 2 University of Gondar, College of Medicine and Health Science, School of Nursing, Gondar, Ethiopia

**Keywords:** Adult, Northeast Ethiopia, Self-medication practice

## Abstract

**Background:**

Self-medication practice (SMP) is the use of medication without the prescription of health care professionals. The major problems associated with self-medication practice have been drug resistance, drug side effects, wastage of resources, and serious health hazards including death. Thus, the main purpose of this study was to assess the prevalence of self-medication practice and its associated factors among adult household members in Meket District, Northeast Ethiopia.

**Methods:**

A community based cross-sectional study was conducted among 722 adult household members in Meket District, from April 5 to May 5, 2017. The systematic random sampling method was used to select study participants. A pre-tested, structured questionnaire was used for data collection using an interviewer-administered technique. Epi-info version and SPSS version 22 were utilized for data entry and analysis, respectively. Univariate and multivariate logistic regression was used to identify association factors.

**Results:**

The overall prevalence of self-medication was found to be 35.9%. Unmarried status (AOR = 2.17, 95% CI = 1.18, 4.01), previous experience of self-medication (AOR = 1.78, 95% CI = 1.22, 2.61), accessibility of pharmacies (AOR = 3.71, 95% CI = 1.31, 10.51), peer/family pressure (AOR = 2.88, 95% CI = 1.98, 4.18) and presence of medication at home (AOR = 1.80, 95% CI = 1.11, 2.92) were factors associated with self-medication practices.

**Conclusion:**

More than one-third of the study participants practiced self-medication. Thus, strengthening communities awareness on drug side effects and integrated efforts of individuals, communities, health facilities, and regulatory bodies are highly necessary.

**Electronic supplementary material:**

The online version of this article (10.1186/s40360-018-0205-6) contains supplementary material, which is available to authorized users.

## Background

Self-medication practice (SMP), as one element of self-care, is the use of medication without the prescription of health care professionals (e.g. resubmitting old prescriptions, sharing medication with relatives/family members or using leftover medications) for the treatment of self-recognized illnesses [1]. Globally, the prevalence of SMP is inconsistent ranging from 32.5 to 81.5% [2–4]. In Ethiopia, the prevalence of self-medication practice ranges from 12.8% to 77.1% [5].

Essential medications are planned to be available inappropriately functioning health systems at intended times, in sufficient amounts, in the proper dosage forms, in assured quality and adequate information, and at prices the individual and the community can afford [6]. However, self-medication practice influences health care seeking behavior of individuals. It leads to wastage of resources, delay in diagnosis of problems and appropriate treatments. Can also lead to serious health hazards and adverse drug reactions [6, 7].

People may practice self medication for a variety of reasons, like the urge for self-care, sympathy for family members in sickness, lack of health services, poverty, ignorance, misbelief, excessive advertisements of drugs, and availability of drugs in establishments other than pharmacies [8].

Self-medication has an important role in health care and in the continued improvement of public education [9]. Despite this studies found out that medical and health science students are the most prone groups to practice self-medication recurrently. The studies done in India in two phases showed that the prevalence of self-medication practices among students were 74.6% and 69.4%, respectively. Oral antibacterial and anti-inflammatory agents and antipyretics were the most common group of drugs used in both phases of the study [10]. Other studies which examined the prevalence of self medication, for example, on students in Belgrade, Serbia [11], three institutes in Nagara, Mandya district, Karnataka [12], South India [13], Jiangsu University, eastern China [14], in Brazil [15], at a tertiary care medical college, West Bengal [16] and Mekele University, Ethiopia [17], reported 79.9%, 92.39%, 78.6%, 47.9%, 56.0%,43.24%, respectively.

Still other studies in primary health care centers in Erbil City (52.6%) [18], a public health care system in Tabuk City (83.3%) [19], Dental patients in Bangalore (100%) [20], at four hospitals in Dar es Salaam, Tanzania (88.6%) [21] and among diabetic patients in Southeast Iran (80.7%) [22], reported the prevalence of self medication practice.

According to such studies, level of education, pervious experience of self-medication, occupation, lack of medical insurance, lack of time to visit physicians, low income [23–25], female sex [26], urban residece [27], youth age [28] and men [29, 30] were factors associated with SMP. Despite the negative consequences of self-medication and its high spread, studies on its prevalence and associated factors in this study area are limited. Therefore, the aim of this study was to assess the prevalence of self-medication practice and associated factors among adult household members in Meket District, Northeast Ethiopia.

## Methods

### Study design and period

A community-based cross sectional study design was used to conduct the study from April, 5 to May, 5, 2017.

### Study area

The study was conducted in Meket district found in North Wollo Zone, Amhara Region Northeast Ethiopia. It is located at 1015 km from Addis Ababa the capital of Ethiopia. This district has 36 kebeles (lowest administrative units) and total of 273,617 inhabitants. Of these, 137,629 (50.3%) were females. In addition to eight government health centers, the district has four clinics and two pharmacies owned privately.

### Source population

All people in Meket district, Northeast Ethiopia.

### Study population

All adults, (age ≥ 18 years) in Meket district, Northeast Ethiopia.

### Sampling frame

The official records were used as frames to determine sample kebeles and households.

### Inclusion criteria

All people aged ≥18 years and had live in for at least six months in the district.

### Exclusion criteria

People who were seriously ill and incapable of hearing and speaking at the time of data collection were excluded.

### Sample size and sampling procedure

The sample size was calculated by using the single population proportion formula (n = [(Zα/2)2 × P (1-P)]/D2), by assumptioning, a prevalence of self-medication practice as 32.55% [31], 95% CI =1.96, margin of error 5%, design effect of 2 and plus 10% non-response rate, the final sample size was 744. In accordance with the multistage stratified sampling method, seven kebeles were selected by using the lottery method. Then, the total sample was proportionally allocated to each kebeles. Finally, participants were selected by the systematic random sampling method.

### Data collection tool and procedure

For the assessment of the theme of the research, a questionnaire which included socio- demographic factors, history of illness in the month preceding the study, and self-medication practice was prepared. The data were collected by 14 health extension workers supervised by seven BSc degree graduate nurses.

Data were collected in a face-to- face interview on a structured questionnaire. Whenever selected houses were found closed or missing adult members, they were revisited continuously for three subsequent days in order to collect data. For house-holds which were missing adult members, the nearest houses were selected, while houses which were found closed during the three subsequent days were considered as non-response cases. When multiple adults were preset in the same household, lottery was used to select a single adult. The required data were collected after written informed consents were obtained (Additional file 1).

### Data quality control

The questionnaire prepared in English was translated into the local language, Amharic and then to ensure consistency, it was retranslated to English. A pre-test was conducted on 38 participants in non-selected kebeles two weeks prior to the actual data collection date. A one day training was given to data collectors and supervisors about the questionnaire and data collection techniques.

### Operational definition

In this study, self-medication practice was defined as any use of medications without the prescribtions of health care professionals (such as physicians, nurses, health officers and other who have government license to prescribe drugs) for the treatment of self- recognized illnesses in the last one month [32].

### Data processing and analysis

Data were cheeked for completeness and consistency before entry. Coded data were entered into EPI-info version 7 and exported to Statistical Package for Social Science (SPSS) version 22 for analysis. Descriptive statistics like, frequency, percentage, mean, standardization, and median were used for data presentation. Univariate and multivariate logistic regression was fitted to identify associated factors with 95% CI using *p*-value < 0.05 as a cutoff point.

## Results

### Socio-demographic characteristics of respondents

In this study, 744 participants were enrolled with a response rate of 722 (97%). The mean age of the respondents was 40.51 years (standard deviation ±14.6). More than half (53.2%) of the participants were female 450 (62.3%) of whom were married. Three hundred and twenty-nine (45.6%) were farmers by occupation. The majority of the respondents 649, (89.9%), were Orthodox Christians and 705 (97.6%) of whom were Amhara by ethnicity. Regarding educational level, 303 (42.0%) were unable to read and write (Table 1).

**Table 1:** Socio-emographic characteristics of respondents, Meket district, Northeast Ethiopia, 2017 (*n* = 722)

Variables	Category	Frequency	Percent
Sex	Female	384	53.2
	Male	338	46.8
Age in year	18–24	92	12.8
	25–34	180	24.9
	35–44	180	24.9
	45–54	113	15.7
	> 55	157	21.7
Marital statues	Married	450	62.3
	Unmarried	105	14.5
	Divorced	102	14.1
	Widowed	65	9.0
Occupation	Farmer	329	45.6
	Housewife	136	18.8
	Civil servant	107	14.8
	Student	71	9.8
	Merchant	65	9.1
	Daily workers	14	1.9
Average monthly household income	< 150 birr	58	8.1
	150–500 birr	292	40.4
	501–1000 birr	143	19.8
	> 1000 birr	229	31.7
Place of residence	Rural	525	72.7
	Urban	197	27.3
Level of education	Unable to read and write	303	42.0
	Only able to read and write	137	19.0
	1–8 grades	105	14.5
	9–12 grades	63	8.7
	Diploma and above	114	15.8
Religion	Orthodox	649	89.9
	Muslim	70	9.7
	Protestant	3	0.4
Ethnicity	Amhara	705	97.6
	Tigray	17	2.4
Number of family	< 5	576	79.8
	5 and above	146	20.2
Relationship with family	Father	283	39.2
	Mother	310	42.9
	Child	115	15.9
	Relative	14	1.9

### Environmental and personal characteristics of participants

In this study, the majority of the participants, 622 (86.15%), traveled over one hour to reach health institutions, while 524 (72.58%) had access to pharmacies. On the whole, 617 (85.46%) had medication at home (Table 2).

**Table 2:** Environmental and personal characteristics of participants, Meket district Northeast Ethiopia, 2017 (*n* = 722)

Variable	Category	Frequency	Percent
Distance of health institution	< 1 h	100	13.9
	≥ 1 h	622	86.2
Accessibility of pharmacy	Yes	198	27.4
	No	524	72.6
Presence of health profession in the family	Yes	56	7.2
	No	666	92.8
Presence of medication at home	Yes	105	14.5
	No	617	85.5
Previous experience of SMP	Yes	402	55.7
	No	320	44.3
Peer/family pressure	Yes	226	31.3
	No	496	68.7
Member of health insurance	Yes	448	62.0
	No	274	38.0

### Reasons of participants for self-medication practice

Perceptions of illness as mild 130 (50.19%), similarity of symptoms with previous illnesses 36 (13.9%) and inability to afford health care fee 33 (12.74%) were the major reasons for self-medication practice (Table 3).

**Table 3:** Reasons of participants for self-medication practice, Meket district, Northeast Ethiopia, April to May, 2017, (*n* = 259)

Reasons	Frequency	Percent
Perceive illness as mild	130	50.2
Similarity of symptoms with past illness	36	13.9
Need quick relive	20	7.7
Unable to afford the health care fee	33	12.7
Dissatisfaction by health care system service	17	6.6
Long waiting time	23	8.9

### Symptoms’ of illnesses and utilized groups of medications

**Fig. 1:**
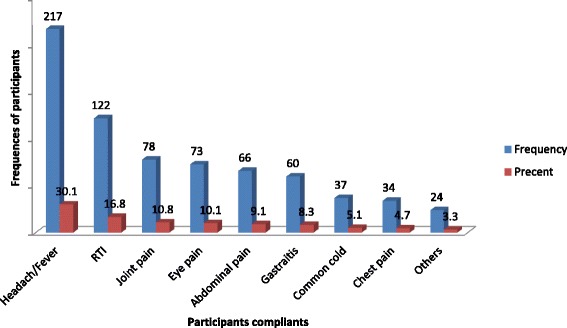
Participants complaints for self medication practice, Meket District, Northeast Ethiopia, 2017, (*n* = 435)

Among the total 722 study participants, 435 (60.2%) complained about illnesses in the last one month. Headache/fever 217 (30.06%) and analgesics/antipyretics 113 (40.79%) were the common symptom and utilized self-medications (Fig. 1).

### Prevalence of self-medication practice

In this study, the prevalence of self-medication practice was found to be 259 (35.9%) (95% Cl: 32.7, 39.2).

### Factors associated with self-medication practice

According to the multivariate logistic regression analysis, unmarried status, previous experience of self-medication, accessibility of pharmacy, peer/family pressure, and the presence of medication at home were factors associated with self-medication practice. Unmarried respondents were 2 times (AOR = 2.17, 95% CI = 1.18, 4.01) more likely to practice self-medication compared to married respondents.

Previous experience of SMP was nearly two times (AOR = 1.78, 95% CI = 1.22, 2.61) more likely experienced self-medication practice than their counter respondents. Accessibility of pharmacy were nearly four times (AOR = 3.71, 95% CI = 1.31, 10.51) more likely experienced self-medication practice compared to those in-accessibility. Respondents who had medication at home were nearly 2 times (AOR = 1.80, 95% CI = 1.11, 2.92) more likely to practice self-medication compared to those who had not such access. The odds ratio of self medication practice among respondents who had peer/family pressure was almost 3 times (AOR = 2.88, 95% Cl = 1.98, 4.18) more likely practice self medication compared to those who had not peer pressure (Table 4).

**Table 4:** Univariate and multivariate logistic regression analysis of self medication practice among participants, Meket district, Northeast Ethiopia, 2017(*n* = 722)

Variables	Category	Self medication practice		COR (95% CI)	AOR (95% CI)
		Yes	No		
Sex	Female	153	231	1.45(1.07, 1.97)^*^	1.11(0.76, 1.63)
	Male	106	232	1	1
Age in year	18–24	32	60	1	1
	25–34	51	129	0.74(0.43, 1.27)	0.86(0.45, 1.66)
	35–44	60	120	0.94 (0.58, 1.52)	1.29(0.62, 2.69)
	45–54	44	69	1.2(0.68, 2.12)	1.42(0.64, 3.17)
	> 55	72	85	1.54(0.93, 2.79)	1.73(0.80, 3.75)
Marital status	Married	140	310	1	1
	Unmarried	42	63	1.48(0.95, 2.29)	2.17(1.18, 4.01)^**^
	Divorced	44	58	1.68(1.08, 2.61)^*^	1.28(0.76, 2.14)
	Widowed	33	32	2.28(1.35, 3.86)^*^	1.32(0.704, 2.48)
Level of education	Unable to read and write	124	179	1	1
	Only able to read and write	41	96	0.62(0.40, 0.95)	0.73(0.45, 1.20)
	1-8grade	34	71	0.69(0.43, 1.10)	0.82(0.45, 1.48)
	9-12grade	20	43	0.67(0.38, 1.2)	0.55(0.25, 1.23)
	Diploma and above	40	74	0.78(0.50, 1.23)	0.85(0.42, 1.71)
Average household monthly income	<150birr	27	31	1.62(0.91, 2.91)	1.28(0.62, 2.66)
	150-500birr	109	183	1.11(0.77, 1.59)	1.22(0.75, 2.01)
	501–1000 birr	43	100	0.80(0.51, 1.26)	0.91(0.52, 1.58)
	> 1000 birr	80	149	1	1
Place of residence	Urban	82	115	1	1
	Rural	117	348	0.47(0.51, 0.99)^*^	1.97(0.69, 5.64)
Accessibility of pharmacy	Yes	89	108	1.72(1.23, 2.41)^*^	3.71(1.31, 10.51)^**^
	No	170	355	1	1
Presence of medication at home	Yes	58	47	2.56(1.68, 3.69)^*^	1.80(1.11, 2.92)^**^
	No	201	416	1	1
Previous experience of SMP	Yes	185	217	2.83(2.05, 3.93)^*^	1.78(1.22, 2.61)^**^
	No	74	246	1	1
Peer/family pressure	Yes	128	98	3.64(2.62, 5.06)^*^	2.88(1.98, 4.13)^**^
	No	131	365	1	1
Due to lack of time	Yes	32	31	1.96(1.17, 3.30)^*^	1.61(0.90, 2.89)
	No	227	432	1	1
Distance of health institution	< 1 h	27	73	1	1
	≥ 1 h	232	390	1.61(1.01, 2.57)^*^	1.45(0.85, 2.47)

^*^Variables those were significant during univariate logistic analysis at *P* value < = 0.05

^**^Variables that was found to have significant association during multivariate analysis at *p* value < = 0.05

## Discussion

In this study, the overall prevalence of self-medication practice was found to be 35.9% (95% Cl; 32.70, 39.20). This finding is in-line with other studies of carried out in different parts of Ethiopia such as Nekemte (36.7%) [33], DireDawa (41%) and Koladiba [31]. This result is also similar with study carried out in South India (35.9%) [34].

The current finding is higher than those of the studies done in Bahir Dar (23,3%) [35], Sire town (27.16%) [36], Silte zone (24.40%) [37] and Brazil (16%) [26]. On the other hand, the prevalence of SMP in this study is lower than those of conducted in Pakistan (61.20%) [38], India (55%) [30], and Italy (69.20%) [32]. This may be due to the differences in socio-demographic factors and sample sizes. In the Pakistan study the sample size was 500 from which health professionals who were found in the community were excluded from the study. In the Indian study, the sample size was 260, and the study participants were only urban residents. Regarding associated factors, unmarried participants were two times (AOR 2.17, 95% Cl; 1.18, 4.01) more likely to utilize self medication compared to married respondents. This is consistent with the study finding in India [34]. The possible explanation could be that unmarried respondents are more influenced by peer pressure than married ones.

Those participants who had access to pharmacies were nearly 4 times (AOR 3.71, 95% Cl; 1.31, 10.51) more likely to utiliz self medication compared to those who hadn’t. This result is consistent with those of studies carried out in China [39], India [34], and Nigeria [40]. This might be due to lack of income and time to consult health care professionals. This is supported by finding in that inability to afford health care fees is noted as the reason for self-medication practice. Previous experience of SMP was nearly 2 times (AOR 1.78, 95% Cl; 1.22, 2.61) more likely to lead to self-medication compared to those who hadn’t previous experience. This study is similar with the study finding in Jimma [41], Uganda [42], Nigeria [40] and Congo [43]. The possible explanation could be that those who had experience may have some awareness about treatment which encourage them, to use self medication rather than taking other actions.

Participants who had peer/family pressure were nearly 3 times (AOR 2.88, 95%Cl; 1.98, 4.18) more likely to use self-medication compared to those who hadn’t. The current result is consistent with those of studies carried out in China [39] and Uganda [42]. The possible explanation may be that friends/families were the common sources of information about medication.

Participants who had medication at home were nearly 2 times (AOR 1.80, 95% Cl; 1.11, 2.92) more likely to use self medication than who hadn’t. This finding is consistent with that of the studies in South India [34] and Egypt [44]. This is due to the fact that participants who store medication mostly for the purpose of emergency in cases of similar illnesses in the future would have the possibility to use it. In the current study, 105 (14.5%) participants had medication at home, and 55.2% of them used self-medication.

The category of medication mostly used in this study included analgesics/antipyretics, antibiotics, GI medications, and eye medication, while headaches, fever, respiratory tract infection, gastrointestinal diseases and eye diseases were the conditions for which self-medication was used. These findings are consistent with other studies [31, 36, 37, 45] as well as studies in Nigeria [40] and India [46].

The most common reasons why respondents practiced self-medication were perceiving illnesses as mild 68 (26.3%), the similarity of illnesses with the past 26 (10.%) and unable to afford health care fee 20 (7.7%).This is similar with studies done in Sire town [36], Jimma town [29] and Iran [47].

As to the limitation; this study was intended to assess modern self-medication practice, which does not include the traditional medications. So, the prevalence of SMP might be under estimated. This imposed the generalization of the findings to all types of medications.

## Conclusion

More than one-third of the study participants practiced self-medication. Factors like being unmarried, presence of medication at home, accessibility of pharmacies, peer pressure and previous experience of SMP were the predictors of self-medication practice. Strengthening of the communities awarness on the side effects of self medication practice and regulation of pharmacies are very important mechanisms to decrease the practice. Thus, integrated efforts of individuals, communities, health facilities, and the regulatory bodies are highly important.

## Additional file

Additional file 1: The aim of the study is to assess Self medication practice and its associated factors among adult household members in Meket District, Northeast Ethiopia. The questionnaire was prepared in English by intensively searching related literatures. It was translated into the local language, Amharic and then to ensure consistency, it was retranslated to English. It included socio- demographic factors, history of illness in the month preceding the study, and self-medication practice. (DOCX 22 kb)

## Abbreviations

AOR: Adjusted odd ratio
COR: Crude odd ratio
POM: Prescription only medication
SMP: Self medication practice
SPSS: Statistical Package for Social Sciences
WHO: World Health Organization
