# Supplementary material for: Self-medication practice and associated factors among adult household members in Meket district, Northeast Ethiopia, 2017
Source: BMC Pharmacol Toxicol. 2018 Apr 10;19:15. doi: 10.1186/s40360-018-0205-6 (PMC5894137; doi:10.1186/s40360-018-0205-6)
Supplement: Supplementary file 1 — The aim of the study is to assess Self medication practice and its associated factors among adult household members in Meket District, Northeast Ethiopia. The questionnaire was prepared in English by intensively searching related literatures. It was translated into the local language, Amharic and then to ensure consistency, it was retranslated to English. It included socio- demographic factors, history of illness in the month preceding the study, and self-medication practice. (DOCX 22 kb) [file 40360_2018_205_MOESM1_ESM.docx]

### Questionnaire English language version

This questionnaire is prepared to be filled by head of the household, if not present any people (Age ≥18 years) who responsible for the household, living in Meket District at least six month, if more than one adult in one household, one person will be selected by lottery method.

Hello, my name is________ and I am going to conduct an interview with you on behalf of __________.You are just invited to participate in a research study to be conducted by __________ at _______________.Please read the following statements and ask any unclear questions before you agree to participate.

Thank you for allowing us to share your precious time and for your willingness to participate in this study. The objectives of this study to assess self medication practice and associated factors towards prevention of negative effect of SMP among households in Meket District and you are chosen to participate in this study.

In order to attain effectively the goal, we are asking you for your generous help. We would like to assure you that privacy will be strictly maintained throughout. There is no need to put your name on the format. No individual response will be reported. It is your full right to participate or refuse in the study. If you don’t want to participate in the study you have full right to refuse. But your honest participation will have a great contribution. So please take a few minute to answer these questions.

Do you wish to participate in the study?

………Yes, I want to participate in the study (please go to the next page)

……….No, I don’t want to participate

Thank you

- Questionnaire number___________________

- Household identification__________________

- Region: Amhara Region, North Wollo Zone, Meket District

- Kebele _____________________

- Interviewer name_______________________

- Date of interview (day/Month/Year)_______

**Part one: Socio demographic variables**

|  | Variables | Code | Remark |
| --- | --- | --- | --- |
| 1 | Sex | 1.Female  2.Male |  |
| 2 | Age | ………………years |  |
| 3 | Marital status | 1. Married  2 unmarried  3. Divorced  4. Widowed |  |
| 4 | Occupation | 1. Farmer 2. housewives  3. Civil servant 4. Student  5. Merchant 6. If others specify……… |  |
| 5 | Average household monthly income  Type of crops and amount  1.…………..  2…………..  3……………. | …………..Birr |  |
| 6 | Are you a member of health insurance? | 1. Yes 2. No |  |
| 7 | Place of residence | 1. Urban 2. Rural |  |
| 8 | Educational level | 1. Unable to read and write 2. Only able to read and write 3. 1-8 Grade 4. 9-12 Grade 4. Diploma and above |  |
| 9 | Religion | A. Orthodox B. Muslim C. Protestant  D. If other specify it |  |
| 10 | Ethnicity | A. Amhara B. Oromo  C. Tigray D. If other specify it |  |
| 11 | Relationships with the family | A. Father B. Mother  C. Child D. Relative |  |
| 12 | Presence of health profession in the family | 1.Yes 2.No |  |
| 13 | Number of family | ………………. |  |

**Part two: personal factors and disease conditions**

| 14 | Are you have any history of illness in the last one month | 1. Yes 2. No | If No skip toQNo27 |
| --- | --- | --- | --- |
| 15 | If yes, what were the health symptoms of the illness  **More than one answer is possible.** | 1. Fever 2. Headache  3. Chest pain 4. Eye pain  5. joint Pain 6.Cough  7.Abdominal pain 8.Gastritis  9.Resparatory tract infection(RTI)  10.common cold  11. If Other (Specify)----------------- |  |
| 16 | Did you use modern medication SMP to relief your symptom? | 1. Yes 2. No | If No skip to QNo26 |
| 17 | If yes, What was the medication that you take to relief your symptom? | 1. Analgesic/Antipyretic  2. GI medication 3. Anti-acid  4. Eye medication 5.RTI treatment 6. Anti-malaria  6. If other specify….. |  |
| 18 | Who is the sources of information to use SMP | 1.Pharmacists 2.Relatives/neighbors  3. Family members 4.from Books or leaflets 5.If other, specify……… |  |
| 19 | Where you get the medication | 1. Pharmacy 2.Friends/neighbors  3.Sharing with family members  4.Left over medication from pervious prescription 5.If other specify |  |
| 20 | How many times per day | 1. One times per day 2. two times per day  3. Three times per day 4. more than three |  |
| 21 | For how long you use | 1.One Week and below  2.Two Week 3.more than two week |  |
| 22 | Why you use SMP rather to go health facility  **More than one answer is possible** | 1. perception of illness as mild  2. Similarity of illness with the past illness  3. unable to afford the health carefree  4. Dissatisfaction by health care service  5. Long waiting time in the health facility  6. To need Quick Relief  7. If other specify…….. |  |
| 23 | Do you check expiry date of medication during purchasing or before using | 1. Yes 2. No |  |
| 24 | What is the outcome of SMP | 1.Improve 2.No change  3. Worse symptom 4.If other specify……. |  |
| 25 | If the outcome is not improve what do you do | 1.Go to government health facility  2.Go to private health facility  3. Use another SMP  4. use traditional medication  5. If other specify……………… |  |
| 26 | If No QNo16 what actions taken to control your symptom | 1. Go to government health facility 2. Go to private health facility 3. Use traditional medication 4. No action taken |  |
| 27 | Have you previous experience of SMP | 1. Yes 2.No |  |
| 28 | Due to lack of time did you have SMP | 1.Yes 2.No |  |
| 29 | Are you thinking it is good practice? | 1. Yes 2. No |  |

**Part three፡ Environmental factors**

| 30 | Accessibility of pharmacy in your environment | Yes 2,No |  |
| --- | --- | --- | --- |
| 31 | How many hours(minuets) take from your home to health institution | ……………………. |  |
| 32 | Do you have currently any modern medication at home | 1.Yes 2.No |  |
| 33 | If yes, tell medication name or show medication |  |  |
| 34 | Why store medication at home | 1.For emergency use  2.For similar symptom occur in the future  3. if other specify |  |
| 35 | Have you peer/family pressure to use SMP | 1.Yes 2.No |  |

Thank you for your participation
